# Determining Spatial Responses of Fishers (*Pekania Pennanti*) to Mechanical Treatments of Forest Stands for Fuel Reduction

**DOI:** 10.3390/ani15030434

**Published:** 2025-02-04

**Authors:** Tessa R. Smith, Eric M. Gese, R. David Clayton, Patricia A. Terletzky, Kathryn L. Purcell, Craig M. Thompson

**Affiliations:** 1 Department of Wildland Resources, Utah State University, Logan, UT 84322, USA; tessa.smith@usda.gov (T.R.S.); pat.terletzky@usu.edu (P.A.T.); 2 U.S. Forest Service, Rogue River-Siskiyou National Forest, Medford, OR 97501, USA; richard.clayton@usda.gov; 3 U.S. Forest Service, Pacific Southwest Research Station, Coarsegold, CA 93614, USA; kathryn.purcell@usda.gov; 4 U.S. Forest Service, Pacific Southwest Region, Vallejo, CA 59804, USA; craig.thompson@usda.gov

**Keywords:** behavior, fisher, forest, *Pekania pennanti*, silviculture, timber, treatments

## Abstract

Historical forestry practices (e.g., fire suppression, heavy timber logging) have contributed to a discernable change in stand composition of western forests in the U.S., which now comprise a tinderbox mixture of increased surface and ladder fuels, dense stands, and fire-intolerant species. Forest managers are mitigating this concern by implementing silviculture practices (e.g., selective logging, thinning, prescribed burning) to reduce fuel loads and improve stand resiliency. Concern for habitat specialists, such as the fisher (*Pekania pennanti*), have arisen as they may be negatively influenced in the short-term by modifications to their environment that are needed to ensure long-term habitat persistence. To address this issue, we initiated an 8-year study in 2010 in Ashland, Oregon, to determine the behavioral response of fishers to fuel reduction treatments applied in forested stands. We measured the distance of each location from eight GPS-collared fishers to all treatments before and after they were treated within each home range, and performed three statistical tests for robustness, including a multi-response permutation procedure, chi-squared test of independence, and a Kolmogorov–Smirnov assessment. We found high variation among individuals to the tolerance of habitat manipulation. Using effect size to interpret the magnitude of fisher response to pre- and post-treatment effects, 1 fisher showed a moderate negative relationship to fuel reduction treatments, 5 exhibited a weak negative response, and 2 had a weak positive association with treatments. We used analysis of variance on the three fishers exhibiting the largest effect sizes to treatment disturbance, and used treatment, temporal, and habitat covariates to explore whether these factors influenced behavioral differences. Treatment season and vegetation class were important factors influencing response distance in the pre-treatment period. Post-treatment variables eliciting a negative treatment response were treatment season and treatment size, and results were slightly different when parsing out individual effects compared to a pooled sample set. Our findings suggested that seasonal timing and the location of management activities could influence fisher movement throughout their home range, but it was largely context-dependent based on the perceived risks or benefits to individuals.

## Simple Summary

The fisher (*Pekania pennanti*) is a medium-sized carnivore found in mature forest stands across parts of the western and northern United States. Although historically abundant in the western U.S., fisher populations declined rapidly after fur trapping, extensive logging, and urban development reduced their numbers. Currently, biologists are concerned about the effects timber harvest practices have on fisher tolerance and adaptability when faced with changes to high-quality habitat stands. Tree removal and the thinning of understory vegetation are frequently used to alleviate the spread of wildfires in previously dense forest stands with a potential for large-scale habitat loss. Yet, a deficit of large trees and important vegetation attributes could be detrimental to fisher survival. We examined the impacts of timber treatments on fisher behavior in a watershed system near Ashland, Oregon, between 2010 and 2017. We assessed where fishers were found in their home ranges before and after treatments occurred (i.e., measuring fisher distance to treatment units). Our results indicated that although most fishers moved away from treated areas, they still used untreated portions of their home ranges. We concluded that fishers were able to tolerate ongoing treatments in their home ranges as long as adequate canopy cover and large structures remained for their use on the landscape.

## 1. Introduction

The fisher (*Pekania pennanti*) is primarily associated with late-successional forests in the western United States [1,2,3]. A few common attributes in these mature forest communities include a mixture of conifer and deciduous trees, multiple canopy layers, high woody biomass, and a complex arrangement of vertical structures [4]. Fishers use an assortment of physical features at variable scales for different activities. For example, at the forest-level scale, successful home range establishment occurs where large-diameter trees, standing snags, riparian drainages, and continuous overhead cover are spread throughout the landscape [1,4,5,6]. On a finer scale, fishers use homogenous stands within their home range, which offer foraging opportunities, rest areas, and travel corridors to habitat patches with similar resources [7]. Finally, on a microsite level, fishers choose specific structures within habitat patches that provides security during resting, denning, or evading predators. Often, fishers are located in distinctive structures that take decades to form, such as hollow tree cavities, broad tree limb platforms, or mistletoe broom clusters in large trees [8,9]. All features are vital to the sustainability of fisher populations in the western U.S.; however, when continuously distributed, they also contribute to increased fire behavior, and management objectives removing or modifying critical habitat properties may conflict with fisher conservation efforts [10]. As such, recommendations are needed to assess the availability and distribution of habitat structures essential for fisher survival and their behavioral response to forest restoration practices.

The fisher population range has contracted in the U.S. and currently consists of only a small portion of its former extent [11,12]. In the early 1900s, the overharvest of fishers extirpated many western populations [13,14]. Concurrently, widespread logging operations fragmented habitat, resulting in fewer dispersal events and loss of genetic diversity [5]. Although fur trapping is currently regulated or banned in the western U.S. [15], genetic diversity and population growth remain lower than expected, leading biologists to speculate whether suboptimal habitat and human disturbances are the principal factors now limiting fisher demographics [16,17]. In particular, the forest composition across fisher ranges has changed considerably in the last century due to the combination of extensive timber extraction and fire suppression [18]. Late-successional forests now comprise smaller-diameter trees, a reduction in coarse woody debris, and a limited diversity of understory flora. These younger forests contain few fire-adapted species, are more homogenous in stand-age class and composition, and accumulate high volumes of understory surface fuels (e.g., twigs, leaf litter); when combined, these elements contribute to the rapid spread of crown fires [19]. These current landscape conditions are the remnants of historical practices and management policy that now produce an elevated risk of frequent, severe wildfires in the western US. Mitigation to resolve this problem involves managers advocating for a more fire-resilient ecosystem using fuel reduction methods. Nevertheless, these activities may remove some critical habitat requirements, such as continuous canopy cover or large trees, that fishers use to hunt, travel, rest, and reproduce.

Our study was conducted in southwestern Oregon as part of the Ashland Forest Resiliency (AFR) project. The main objective of the AFR project was to create fire-adapted stands by reducing surface and ladder fuels, ultimately producing forest stands with resilient fire suppression conditions. To do this, thinning and prescribed burns were implemented, which used together has been shown to successfully slow the rate of fire spread and repress crown scorch in severe fire situations [20,21,22]. These prescription treatments are necessary to modify fire behavior, but we wanted to ascertain whether simplifying the stand structure had potential consequences on space use by fishers.

Recent studies have focused on how silviculture methods and wildfire affect the behavior of fishers occupying late seral forests [23]. For example, a monitoring project in the Sierra National Forest, California, concluded that fisher occupancy and annual survival were lower in areas with fuel reduction treatments [24]. However, research assessing the direct impacts of restoration activities (e.g., thinning, burning) on important habitat components concluded that fishers may tolerate ground-disturbing events temporarily, depending on the extent or intensity of the treatment [18,25]. Furthermore, research using theoretical models assessed how habitat quality changed after varying levels of stochastic events, both natural and man-made. The results of these models inferred that temporary trade-offs in local habitat loss from restoration activities warranted consideration as it would reduce the possibility of more widespread ecological damage from a stand-replacing wildfire [26,27].

Nevertheless, little work has been completed in determining whether fisher behavior, at the individual level, changes in a before/after context due to disturbance events, or whether vegetation removal as a result of silviculture activity will affect which structural features fishers use within their home range. Our first objective was to examine whether the effects of mechanical fuel treatments discouraged fisher activity near treatment areas. We hypothesized that before treatments occurred (pre-treatment), fishers would use their entire home range, including the intended treatment areas. After treatments were applied (post treatment), we expected them to avoid commercially treated areas up to a particular threshold distance; however, they would continue to move through the noncommercial treatment units within their home range. One study described commercially treated stands as sites where large, healthy trees were removed for economic profit, resulting in a reduction in canopy density [28]. In the AFR project, variable density management was employed as a commercial treatment method. This activity removed trees <43 cm in diameter to increase the spacing between mature trees and reduce canopy cover to a minimum of 60%. In contrast, noncommercial treatments were less intensive; the high crown canopy was left untouched, but the understory was thinned by extracting shrubs, downed wood, and small saplings of no economic value. Both methods affected overhead cover at different levels and intensities, and a decline in any canopy shelter may affect wildlife distributions in a spatial or temporal context. For fishers in particular, dense canopy cover was the principal habitat element required when fishers dispersed or established home ranges [25,29]. Therefore, in addition to exploring the relationship between the positional distances of fisher to treated units, we further examined what biological and anthropogenic variables (e.g., canopy cover or treatment type) might drive fisher movement towards or away from a treatment action.

## 2. Materials and Methods

### 2.1. Study Area

**Figure 1:**
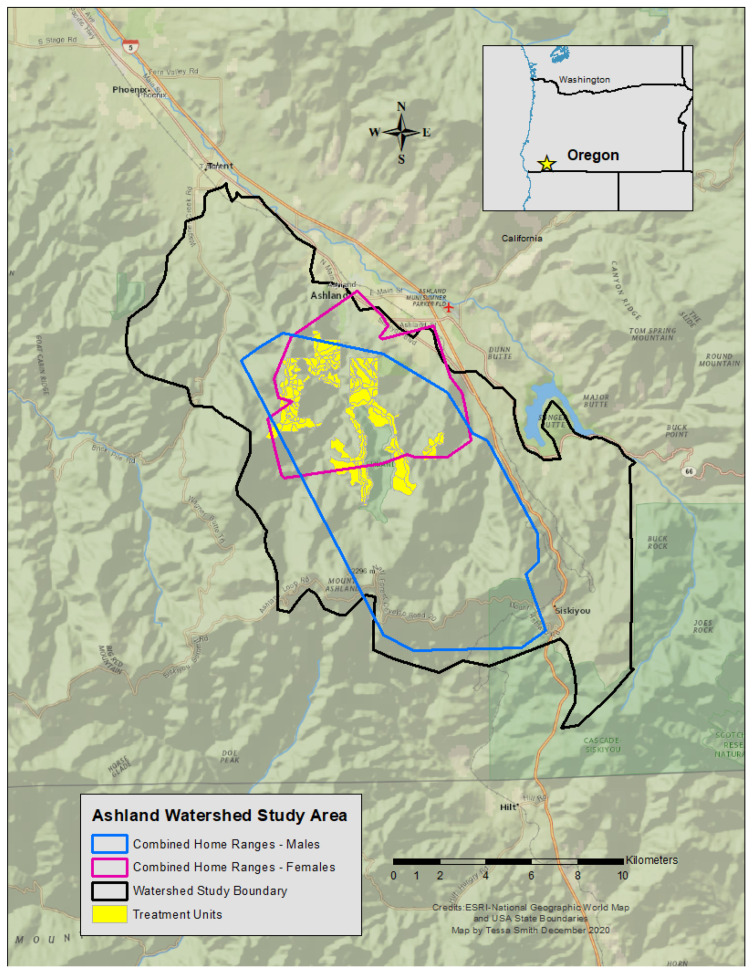
Map of the Ashland Watershed Study Area, Oregon, 2010–2017.

The study area was in a municipal watershed near Ashland, Oregon, USA (Figure 1), where multiple federal and private entities co-manage the area. The 6300 ha site sits within the Rogue River-Siskiyou and Klamath National Forests, and consists of variable topography across the study site. Elevations range from ~600 to 2000 m above sea level. Its climate is Mediterranean with warm, dry summers, and cool, wet winters. Habitat classes are characterized by plant associations, in which vegetation communities are organized by dominant conifer species (e.g., Douglas-fir [*Pseudotsuga menziesii*], ponderosa pine [*Pinus ponderosa*]), hardwood species (e.g., Pacific madrone [*Arbutus menziesii*], California black oak [*Quercus kelloggii*]), or understory species (e.g., ocean spray [*Holodiscus discolor*], manzanita [*Arctostaphylos* spp.]) [30].

Timber management has been limited in the area over the last century. Remnants of past clear cuts are barely visible on the outer edges of the watershed boundary, whereas small-scale timber harvests scattered throughout the interior have resulted in stand succession in various stages [31]. While historical logging practices have contributed to shaping forest structure and complexity, the primary mechanistic force for landscape change is wildfires. Fire return intervals for the region range from 5 to 75 years; however, fire regimes are difficult to assess in the Siskiyou Mountain range because of the distinctive vegetation and topographic variation [30].

### 2.2. Capture, Handling, and Monitoring

Fishers were captured, radio-tagged, and tracked within the Ashland watershed boundary between 2010 and 2017 in fall and winter seasons, with no trapping conducted during the sensitive reproductive period of spring and summer seasons. In each trapping period, new and recaptured individuals received a new GPS-collar that was tested for functionality and full battery capacity. For recaptured individuals, we replaced old collars with a new collar when the battery life had declined, the battery had died, or the collar had malfunctioned (e.g., collar produced erroneous locations or failed to acquire locations). We also fit a new collar on recaptured fishers that had slipped their collar in the interim between trapping seasons. We used live traps (Model 108, Tomahawk Live Trap, Hazelhurst, WI, USA) with a modified cubby box attached to the cage [32,33]. We baited traps using raw chicken supplemented with an assortment of scent lures on the bait. Long-distance call lures were applied to several trees to serve as broadcast scent signals. All methods for animal handling were in accordance with guidelines set by the American Society of Mammologists [34].

Once captured, fishers were anesthetized with a mixture of ketamine (22.5 mg/kg) and diazepam or midazolam (0.125 mg/kg) administered via hand syringe. We inserted a passive integrated transponder (PIT) tag (Biomark, Boise, ID, USA) sub-dermally to uniquely mark individuals. Fishers were sexed, and then they were aged based on sagittal crest development, tooth wear, and weight condition into age classes: juvenile, subadult, or adult [9,35]. We fitted adult fishers with micro-GPS radio collars (Quantum models, Telemetry Solutions, Concord, CA, USA). Once processing was completed, we released fishers at their original capture location and tracked them 24 h later to confirm successful recovery.

The majority of transmitters were programmed to attempt to fix satellite locations every 10 h on average to extend battery life. We estimated GPS collar accuracy by placing two test collars in contrasting habitats for 48 h and calculating the mean distance between the collar locations and known locations from a handheld GPS device. The monitoring of animals occurred once a week using ground telemetry to determine home range extent and assess survival. Every 3–4 weeks, we tracked GPS-collared individuals within 100 m of the animal’s position and remotely downloaded stored locations. We reduced the temporal autocorrelation for each animal by retaining locations collected ≥10 h apart. We retained all 3D locations, plus 2D locations with Horizontal Dilution of Precision (HDOP) values ≤ 7, preserving sufficient sample sizes and precision quality for analysis [36,37,38].

### 2.3. Home Range Estimates and ArcGIS Analysis

Home range delineations for each fisher were derived using the minimum convex polygon (MCP) method calculated with the program Geospatial Modeling Environment (http://www.spatialecology.com/gme (accessed on 9 June 2017)). We calculated the 100% MCP for each individual fisher. To determine the minimum number of locations required for home range estimates, we found that 25 locations per fisher were needed to reach the asymptote of the area-observation curve for home-range size [39].

Forest treatment polygons were defined for management activity by forestry personnel after treatments were completed. We imported the polygons into ArcGIS 10.3.1 (Environmental Systems Research Institute [ESRI], Redlands, CA, USA) and executed the Clip tool to intersect treatment polygons within each fisher home range. We generated distance measurements using the GPS locations of each fisher to all respective treatment units (hereafter, also referred to as a “unit”) inside the home range. We used distance to the nearest edge of a treatment polygon instead of the centroid since the entire unit was manipulated. Because multiple treatments occurred simultaneously or within short periods of one another, we calculated the distance to each treatment unit instead of the nearest unit to reduce violating assumptions of independence between sampling observations. Next, we separated locations into pre- or post-treatment points with respect to the date of the treatment. Dates acquired from operation managers were limited to a month and year; therefore, data were filtered to exclude animal locations <30 days of a unit being treated. Finally, we spatially joined tables for distance measurements and treatment attributes in ArcGIS.

### 2.4. Statistical Analyses

We focused our inquiry on mechanical treatments, where tree density management and canopy closure reduction were the primary objectives. Histogram distributions for pre- and post-treatment observations for each fisher revealed highly skewed data, so we tested for unequal variances using Levene’s test [40]. Because variances were unequal in the majority of the data, we performed nonparametric tests for statistical analyses. However, we realized that large amounts of data often lead to small *p*-values, indicating significance when it may not exist (i.e., false positive). Therefore, we addressed this predicament by performing 3 different analyses to compare statistically significant results, but did not make conclusions based on *p*-value outcomes, as they can be confounded by their dependence on sample size [41]. Instead, we represented the strength of the relationship between the treatment periods and fisher distances by measuring the effect size, which quantifies the magnitude of the difference between pre- and post-treatment distances.

Because of the small sample size, non-parametric distributions, and data dependence, we conducted 3 different statistical tests to assess the robustness of our results. The Kolmogorov–Smirnov test, multi-response permutation procedure, and chi-squared test of independence were used to compare the distributional response of each fisher to pre- and post-treatment applications, using the Euclidean distance from a treated unit as a proxy for behavioral responses. The Kolmogorov–Smirnov (K-S) test evaluated whether the empirical cumulative distribution functions (ECDFs) of sampled distances pre- and post-treatment period differed from one another [42]. A K-S statistic was calculated based on the maximum vertical distance between the pre- and post-treatment ECDF curves with significance levels (α = alpha) generated using the test statistic, D.

We used the multi-response permutation procedure (MRPP; [43]) to analyze the differences in the means between pre- and post-treatment distances for each fisher. The MRPP test computed pairwise distances between all observations within each group (i.e., pre- or post-treatment distance measurements) and calculated a weighted average for each group [44]. The test generated a test statistic, delta, to determine dissimilarities between the groups. We compared an observed delta to an expected delta to produce an approximate Pearson type III *p* value. Expected deltas were obtained by permuting all possible combinations of each group and then recalculating the weighted average. Significance was determined by evaluating whether dissimilarities existed between the permuted datasets and observed dataset, indicated by an appropriate *p* value statistic (α < 0.05). Although the *p* value determined significant differences in the sampling groups, we also evaluated the effect size of the differences, which would be independent of sample size. A chance-corrected agreement coefficient (A) signified within-group homogeneity using the expected and observed deltas. The A-coefficient was calculated on a scale between 0 and 1; when A = 1, all within-group observations were identical, whereas when A approached zero, dissimilarities existed between groups [43].

The third test was the chi-squared test of independence, which was performed by binning the distances into pre-defined intervals, with short-range distances split into numerous classes and long-range distances lumped into fewer bins. We examined the dataset distributions to determine what distance increments were biologically relevant when related to behavioral responses, using previous research of fisher treatment response as an additional guide in partitioning our bin categories [45,46]. Because we were interested in determining the threshold distance at which fishers would tolerate treatment effects, we focused on splitting our data into smaller bins below the mean in order to capture a defined distance measurement. Chi-squared tests are sensitive to sample size requirements; therefore, we retained appropriate sample sizes (i.e., > 5) within each bin category [47]. We subsequently followed our statistical tests with strength tests to determine the direction and magnitude of significance using effect size as our strength test [47]. Thus, Cramer’s V test from chi-squared results and the A-coefficients from the MRPP test were used to calculate appropriate effect sizes [43,48]. All statistical analyses were implemented using program R [49]. The K-S test, chi-squared test, and effect size computations were run using base R packages, while the ‘mrpp’ procedure was executed using the ‘vegan’ package [50].

To determine whether environmental or treatment factors influenced fisher distance movements in the pre- and post-treatment periods, we extracted site-specific variables from the animal locations and the treatment areas for a post hoc regression analysis. We ran ANOVA models with categorical covariates that included treatment variables (treatment size [very small, small, medium, large, very large], treatment type [commercial, noncommercial]), treatment temporal variables (season [spring, summer, fall, winter], fisher location time of day [morning, afternoon, evening, night]), and floristic variables (canopy cover, habitat classification). We did not include “individual” as a random factor, due to the small sample size of only 3 animals. We included the treatment size and type in the pre-treatment analysis to measure fisher use in those designated areas prior to treatment and then compared their space use following treatment. Treatment size, type, and temporal data were derived from information collected by U.S. Forest Service personnel performing density management practices, while floristic data were obtained using LANDFIRE datasets (www.landfire.gov/vegetation.php (accessed on 10 October 2024)). Canopy cover was binned into 5 classes: open-low (0–19%), low-moderate (20–39%), moderate (40–59%), moderate-high (60–79%), and high (≥80%). For habitat classes, we reclassified similar habitat types into fewer categories for ease of model interpretation. Vegetation categories from the LANDFIRE dataset were placed in 4 classes, with each representative proportion signifying its prevalence in the study area: low elevation shrubland (24%), Sierra mixed conifer (19%), conifer-hardwood (17%), and conifer (40%).

To further explore the potential drivers behind avoidance behavior specifically, we tested a pooled subset of fisher data which exhibited significant findings from our first analyses (i.e., distance response and effect size tests) using individual ANOVA models. The ANOVA models were run with the assumption that some individual variation would be evident in response behavior (i.e., fisher distance) to treatment disturbance. We then performed ANOVA tests on individual fishers from the pooled subset above to compare the within-population response variability of fisher distance to treatment effects.

## 3. Results

### 3.1. Capture and Monitoring

During 2010–2017, we deployed 23 GPS collars on captured fishers, but only 10 individuals had reliable GPS collars that remained on-air with successful fix rates. Average acquisition rates for the GPS collars were 32%. From these 10 fishers, we acquired a total of 1352 GPS locations for analysis, ranging from 24 to 263 points (*x̅* = 135.2 ± 108.36 standard deviation (SD); Table 1). Over this study, we tracked fishers for a total of 925 days (*x̅* = 92.5 ± 75.24 SD), although females accounted for a larger proportion of time followed due to their smaller home ranges and recapture success when replacing failing transmitters. Fisher M02 was monitored for the shortest period of time and his collar acquired the fewest number of locations compared to other individuals (Table 1). However, his GPS locations were equally distributed in the pre- and post-treatment periods, locations were spread throughout his large home range, and total days monitored spanned several months. Therefore, his data were retained to ascertain tolerance behavior given they experienced the largest size and highest number of mechanically treated units within a fisher home range.

### 3.2. Home Ranges and Treatment Units

Home-range size differed between the sexes with a mean female size of 16.27 km^2^ ± 8.87 (*n* = 7) and males averaging 69.29 km^2^ ± 31.19 (*n* = 3), more than four times larger (Wilcoxon test, V = 55, *p* = 0.002). The number of treatments within each fisher home range varied widely and our analysis only considered mechanically altered units (i.e., vegetation removal by machine), which accounted for 60% of all treatments. The remaining 40% of treated areas included pile burns, prescribed burns, and non-mechanical treatment methods, which consisted of removing vegetation by hand (e.g., with a chainsaw or hand saw). Non-mechanical methods were not well quantified or shared by operation managers, and burn plans were often delayed or not implemented until after our study concluded. Thus, we did not include these activities in our analyses. The number and proportion of mechanical treatments per individual home range area varied between 8 and 83 units (8% to 34% of a home range; Table 2). Notably, some of the treatment units coincided with multiple female territories due to the proximity and overlap of home ranges (Figure 1). The percentage of treatments within male home ranges was lower, mainly due to larger territories and the propensity of males to explore areas outside of the prescribed treatment blocks. Most activities across the entire study area comprised mechanical methods in treatment units categorized as either commercial or noncommercial (Table 3). Both commercial and noncommercial treatment units were similar in size (hectares) and number of units treated. When combined, the mechanically treated units totaled 72.7% of the entire area managed for vegetation removal or understory improvement.

### 3.3. Treatment Responses

**Figure 2:**
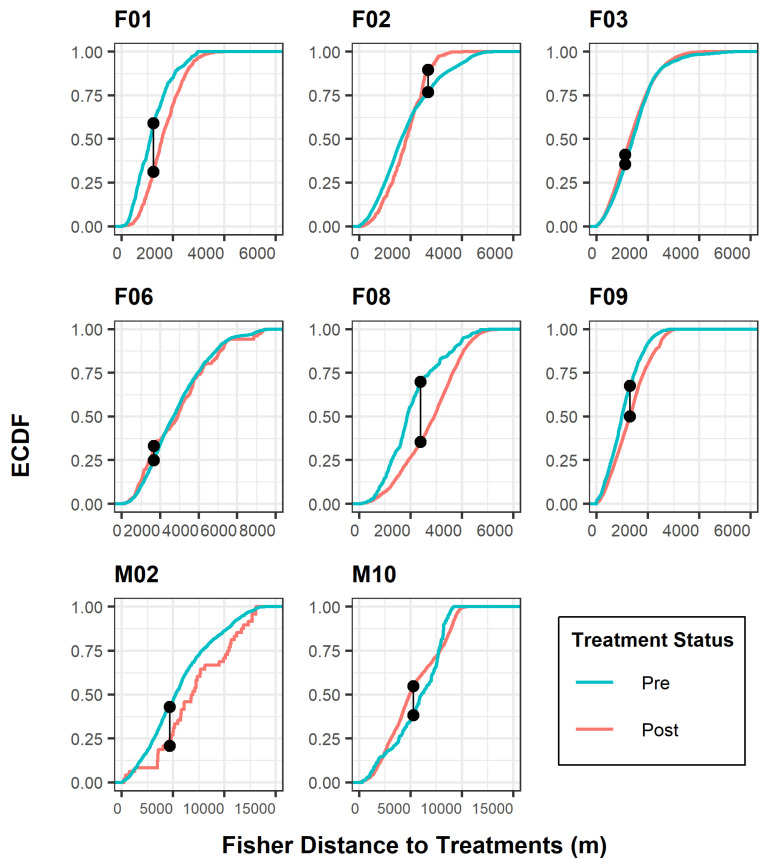
Kolmogorov–Smirnov test for individual fisher response to treatment effects, Ashland Watershed Study Area, Oregon, 2010–2017. ECDF is the empirical cumulative distribution function, defined as the probability distribution of two observed curves. The connected black points indicate where the maximum separation between each distribution curve occurs in relation to the distance (m) from a treated unit. The shifts in distribution lines correspond to tolerance levels for each period. For example, a red curve shifted to the right of a blue curve along the x-axis suggested that fishers were further from treated units following post treatment.

**Figure 3:**
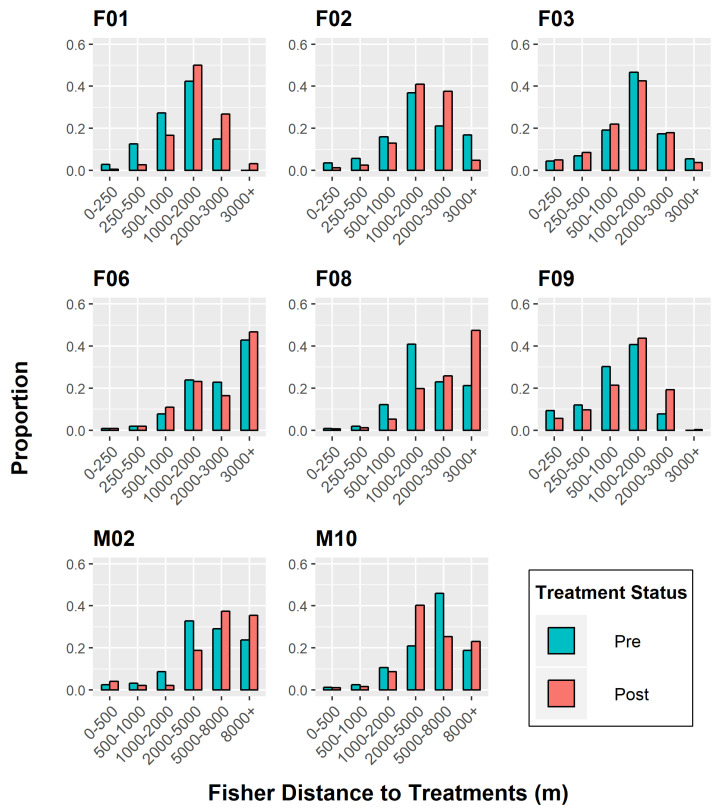
Chi-squared independence test to determine differences in individual fisher response to treatment effects, Ashland Watershed Study Area, Oregon, 2010–2017. At distances below ~1000–2000 m, fishers were found closest to treated units in the pre-treatment period (higher frequency of blue bars). In the post-treatment periods, fishers were found further from treated units, signifying a negative response until a threshold distance was reached at ~2000+ m (higher frequency of red bars). Variable responses were found for F03, F06, and M10.

We did not perform any statistical analyses on two fishers (F04, M04), as both individuals had insufficient location data during the post-treatment phase of this study. For the remaining fishers, we found variability in the distributional frequencies of distance to treatments between the pre- and post-treatment periods. Two analysis tests, K-S and MRPP, resulted in *p*-values <0.05 for all eight fishers of interest (Table 4), indicating significant shifts towards or away from treated units. The chi-squared test of independence also produced significant outcomes (α < 0.05) for all fishers except M02 (Table 4). Overall, six fishers (F01, F02, F06, F08, F09, M02) revealed intolerant behaviors to mechanical treatments, meaning a majority of fishers were located further from treated units following treatment. In contrast, two fishers (F03, M10) displayed movements closer to treatments after mechanical activity was completed (Figure 2 and Figure 3).

The K-S curves (Figure 2) illustrated the relationship between treatment periods and the cumulative frequency of the response variable of fisher distance to treatment. Two important considerations with this test are the maximum distance between curves and the shift in curves relative to distance. Distance between curves denotes whether there is a significant difference between treatment data distributions. Shifts in curves relative to distance explain which treatment period the fisher tolerated best according to the frequency of distances closer to treated areas. Five fishers (F01, F08, F09, M02, M10) exhibited the greatest distance difference between curves, although the K-S analysis resulted in a significant outcome for all fishers. As noted previously, all but two fishers (F03, M10) exhibited intolerant behavior to areas after treatments occurred, indicated by a shift in the K-S post-treatment distance curve to the right (Figure 2).

The chi-squared test of independence mirrored the K-S test patterns, excluding the insignificant outcome of M02’s behavior (Figure 3). Again, F03 and M10 were the only individuals who displayed tolerance to treated units, moving closer to those areas after operations took place. However, their response to treatments was weak in comparison to other individuals and varied across distance categories.

The relative frequencies (i.e., proportion) of post-treatment distances were higher for five fishers (F01, F02, F06, F08, F09) when they were located ≥2 km from restoration areas (Figure 3), regardless of whether the units were commercially altered or not. At this threshold limit, fisher distances were either equivalent between the two periods or showed higher post-treatment proportions. This observation supported our original hypothesis that fishers would avoid treated units up to a particular threshold distance.

**Figure 4:**
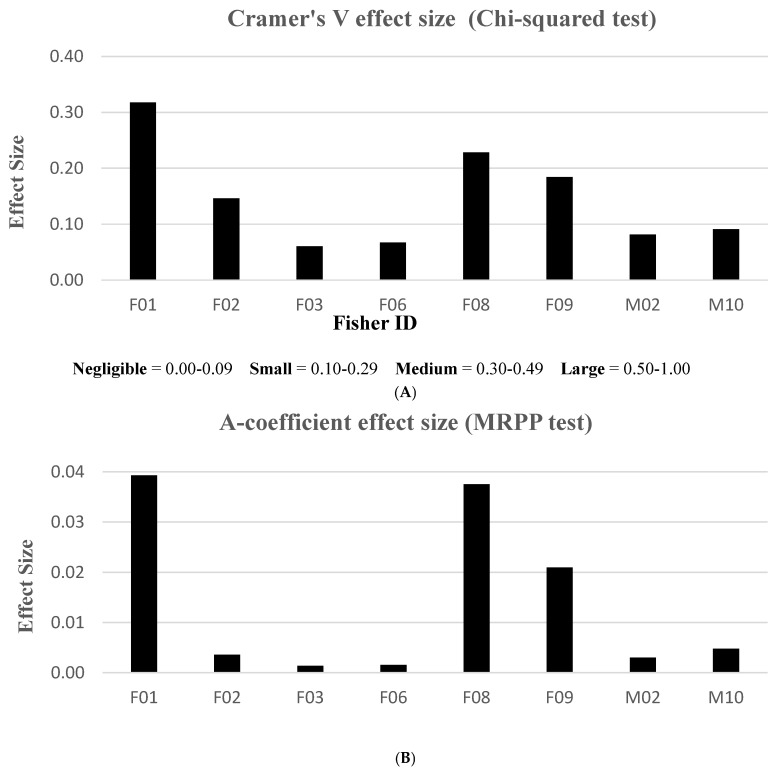
Effect size results measuring the magnitude of each fisher response between pre- and post-treatment periods, Ashland Watershed Study Area, Oregon, 2010–2017. Cramer’s V (**A**) and A-coefficient (**B**) produced similar patterns. An index to interpret effect size is shown for Cramer’s V, but no index was found in the literature for the A-coefficient for the MRPP method.

When evaluating effect size, only the MRPP and chi-squared test statistics resulted in measurable effects, using the A-coefficient and Cramer’s V test, respectively. We were unable to compute effect sizes with the K-S statistic. We elucidated Cramer’s V results using range values classified in “negligible”, “small”, “medium”, or “large” effect size categories. The MRPP method, however, did not have an associated range interpretation for effect size. Thus, graphical results were primarily used to validate similar patterns in Cramer’s V effects. We found that treatments in the home ranges of three fishers (F01, F08, F09) rendered stronger effect size responses compared to the other fishers (Figure 4). According to Cramer’s V descriptive index, no fishers exhibited “large” effects and only one animal (F01) had a measured “medium” effect to treatment activities. Three fishers (F02, F08, F09) revealed “small” effect treatment responses, while the remaining fishers fell into the “negligible” effect category. Although the MRPP and chi-squared analyses resulted in different effect size scales, similar patterns emerged for 7 of 8 fishers tested; the lone exception was F02’s larger discrepancy (Figure 4).

Three fishers (F01, F08, F09) demonstrated the largest effect sizes in our first analysis (Figure 4); therefore, we used these females as our pooled sample dataset for the first ANOVA model (Table 5). Prior to treatment, fisher proximity to the proposed treatment units was influenced by the variables of season, vegetation class, treatment type, treatment size, and canopy cover. The only variable without a significant effect was time of day. Following treatment, proximity was influenced by season, vegetation class, and treatment size. Shared factors that lent a substantial effect on response behavior during both time periods were season, treatment size, and vegetation class.

**Figure 5:**
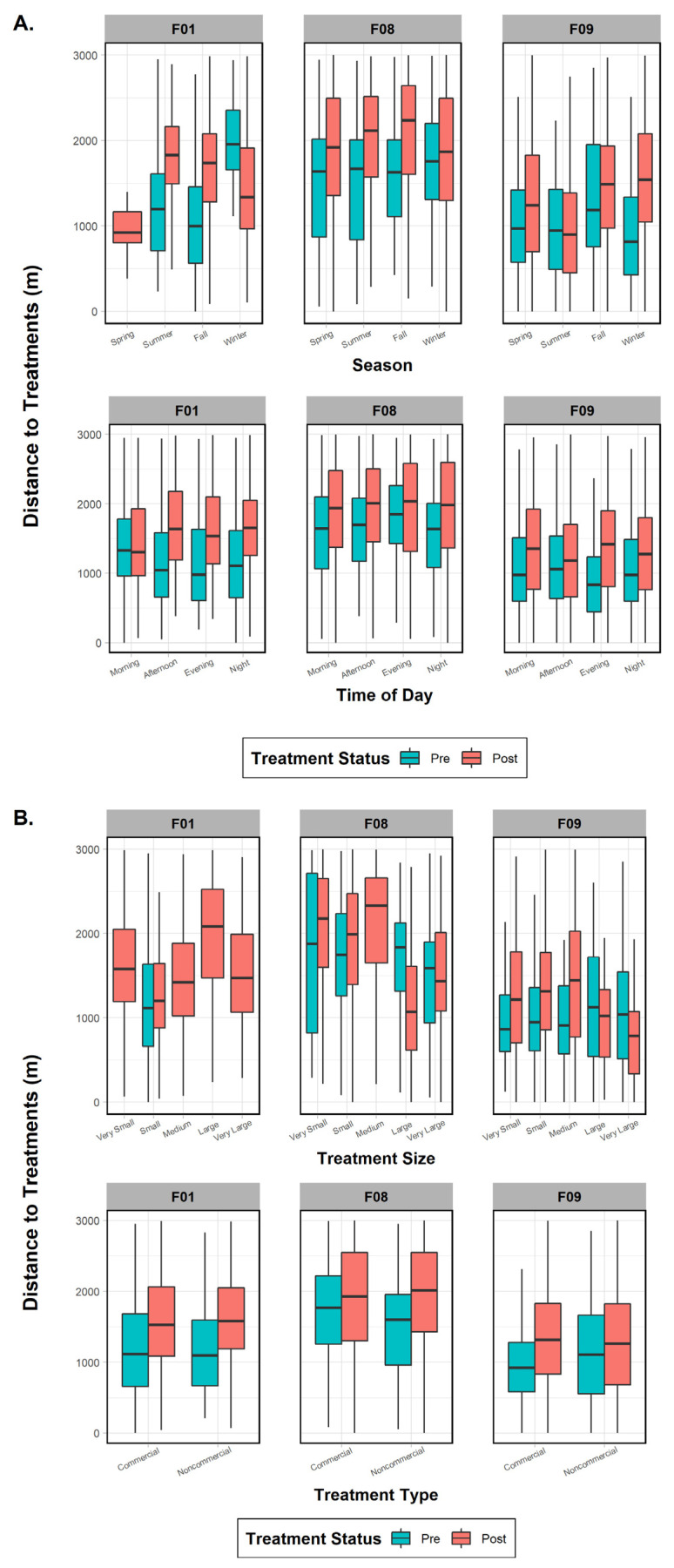

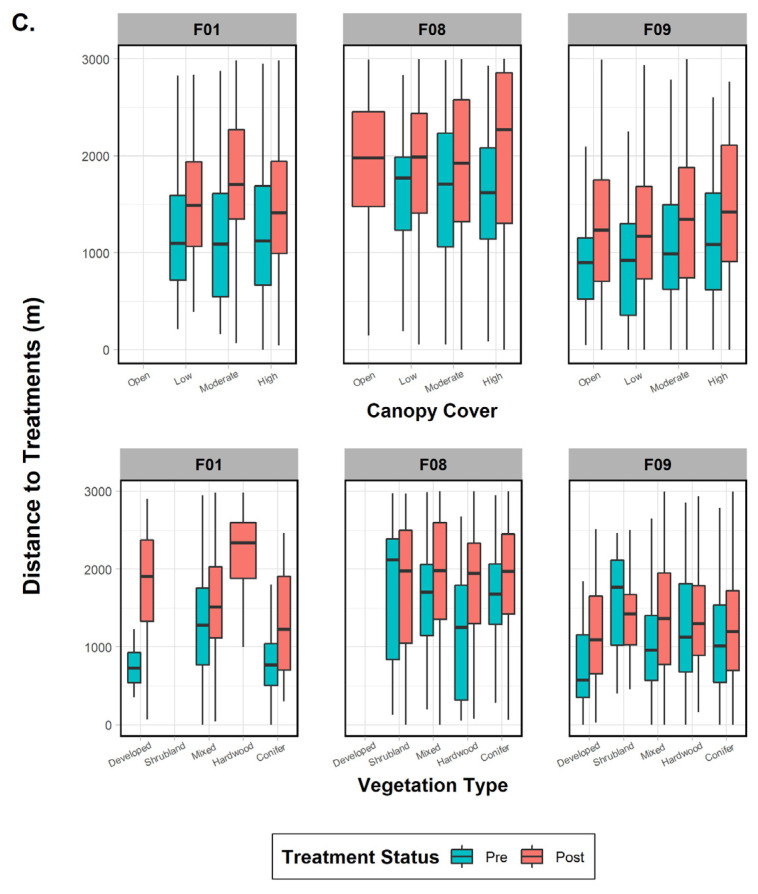
Comparison of mean, range, and error bars of environmental and treatment factors influencing fisher distance to treatments in the pre- and post-treatment periods for fishers F01, F08, and F09, Ashland Watershed Study Area, Oregon, 2010–2017. Variables examined included (**A**) season and time of day, (**B**) treatment size and type, and (**C**) canopy cover and vegetation class. Season, time of day, and vegetation class were significant factors driving fisher response for the pre-treatment period, whereas season, treatment type, and canopy cover were important in the post-treatment period. Changes in the width of the histogram column are an artifact of program R.

The three females were then tested individually to ascertain whether specific factors influenced their behavior to treatment effects and whether any variables were common on a subpopulation scale. One fisher (F01), despite data deficiency in one variable category (treatment size), did not show significant results in the pre- or post-periods for treatment type (commercial versus noncommercial; *p*-values > 0.45), indicating that it was not an influential factor (Figure 5). Season (both *p*-values < 0.001), time of day (both *p*-values < 0.001), and vegetation class (both *p*-values < 0.001) were important in both periods, with the addition of treatment size (*p* < 0.001) and canopy cover (*p* < 0.001) after treatments took place (Figure 5). Results for fisher F08 indicated that all variables (all *p*-values < 0.04) affected her distance behavior in the pre-treatment period (Figure 5). The post-treatment phase results, on the other hand, found that only four variables were significant: season (*p* < 0.001), treatment type (*p* = 0.004), treatment size (*p* < 0.001), and canopy cover (*p* < 0.001). Surprisingly, results for fisher F09 showed that every variable except canopy cover influenced her distance to treatments in both time periods (both *p*-values < 0.001; Figure 5). Finally, when comparing variables between the three females, we found three mutually shared factors in the pre-treatment period that would affect response distance: season, time of day, and vegetation class. Post-treatment variables eliciting a negative response and common to all three fishers were season and treatment size (Figure 5).

## 4. Discussion

The sensitivity of wildlife to anthropogenic change has long been an important question in the scientific community. Human-modified landscapes, ranging from urbanization and development to logging operations and human-caused wildfires, impact the spatial distribution and behavior of a multitude of wildlife taxa [51,52,53,54]. Research has primarily focused on the variable disturbance response of species at the population level, where both positive and negative associations exist. For instance, boreal wolves (*Canis lupus*) in the forests of Quebec, Canada, avoided heavily logged areas [55], whereas those in the Canadian Rockies selected for post-fire logging where foraging opportunities increased [56]. Similarly, wolverines (*Gulo gulo*) were reported to be sensitive to a range of human disruptions, including resource extraction activities [57,58]. However, another study found that wolverines were attracted to cut-block units because of opportunistic food resources, although edges were preferred over the interior of cut-blocks due to predation risks [59]. These examples reference a growing literature describing the variation in population-level responses to human activities across the landscape, but few studies have touched upon the individual responses within a population that can complicate the interpretation of disturbance-related behavior.

We found that fishers responded to anthropogenic stressors on an individual basis, where each animal displayed varying tolerance levels. Unexpectedly, two individuals (F03, M10) exhibited weak, positive reactions to timber management activities occurring within their home range. In contrast, the remaining six fishers displayed varying degrees of aversion to post-treated units. A possible explanation for individual variation could be attributed to different fisher personalities, which would reflect how they spatially distribute themselves on the landscape relative to changing habitat conditions [60]. For example, an individual with a bold personality might express tolerance or perhaps curiosity, when confronted with unfamiliar circumstances and modified habitat conditions. Moreover, if the altered environment provided new exploitable resources and reduced competition, it may attract individuals to those areas. In contrast, animals exhibiting shy or cautious tendencies might perceive risks associated with familiar places changed by human perturbations. If they remain in place but are susceptible to environmental stressors, such as a reduction in critical structures or prey bases, they may under-utilize habitat resources and negatively affect their fitness potential [61]. Furthermore, if the individual shifts its home range as a direct result of habitat alteration, they infringe on conspecific territories and become vulnerable to intraspecific conflict and competition. Typically, these traits, boldness versus caution, are associated with male and female fishers, respectively, particularly with regard to dispersal behavior [62,63]. However, to our knowledge, there has been no effort to understand how this may vary within sexes.

We did not find any fishers permanently shifting their home ranges after treatments occurred. Results indicated that fishers were able to tolerate disturbance-related effects by utilizing other areas of their home range, similar to behavioral responses reported in two other studies [24,46]. In fact, F01, F06, and F08 had adequate space within their respective home ranges to avoid treatment units. A plausible explanation for varying tolerance levels can be found in the home range overlap between several female fishers. Although F03, F06, F08, and F09 had overlapping home ranges, their temporal location points indicated that they rarely came into contact with one another. For example, F03 had treatments scattered in the center of her home range, which overlapped with F06, F08, and F09 to some degree. If competition with other fishers were driving forces, she may have had no choice but to move through treatment units to avoid contact with the other females; thus, her movements suggest a positive response to treatment effects. In contrast, F01 and F08 avoided their treatment areas by utilizing a shared portion of home range overlap where occasional contact occurred. The area of overlap contained no treatment units, and several interactions took place over a year between the females. Possibly, these two fishers were related and, at some time, the juvenile dispersed only a short distance from its natal range. Contact between the offspring and parent, then, may be tolerated as home range establishment progressed. Taken together, these observations suggest that interspecific interactions may be more influential over fisher movement patterns than dispersed management actions.

From a management standpoint, treatment size and configuration, management intensity, and management duration may have influenced differences in fisher behavior. One study reported that fishers tolerated up to 2.6% of their habitat being treated per year [18]. Their study design employed a 14 km^2^ cell unit, equivalent to the size of our average female territory. Because our project comprised higher numbers of treated units (Table 2) compared to the Zielinski study [18], we believed all fishers would avoid post-treated areas. On the contrary, we found that our fishers reacted to vegetation removal by varying degrees, irrespective of the percentage of home range treated. For example, F01’s home range encompassed the fewest treated units and lowest proportion of treated area than any other fisher (Table 2); yet, she exhibited the largest intolerance to treatment effects (Figure 4A,B). Fishers F08 and F09 both demonstrated similar, albeit weaker negative responses; however, their home ranges included more treated units than F01. Interestingly, F03 had a comparable number of treatment units in her home range as F08; yet, she displayed the opposite (weak positive) response effect from all other females.

In addition to percentage of area treated, spatial configuration also appeared to be an important component. In F01’s home range, treated units were concentrated only along one edge, whereas F08 and F09 both had treatments placed near the center of their home ranges, which may have limited their movement through their core territories. Additionally, we noticed that the majority of F01 and F08’s GPS locations were grouped far from the treatment units. Spatially, locations for F01 and F08 were often clustered in areas >1 km away from post-treated units, which resulted in a higher frequency of distance counts farther from managed units, and thus a larger negative response to management activities. Other fishers had locations scattered throughout their home range with fewer clusters, and our analyses showed a smaller effect size to treated units. Despite their intolerant behavior, fisher F01, and to a lesser extent F08, could have utilized the untreated areas of their home ranges adequately in the post-treatment period, thus avoiding the portion of home range area that was mechanically altered.

Similarly, F09 displayed intolerant behavior to mechanical treatments in the post-treatment period as well. Treatments were centrally located in an equivalently sized home range extent as F01 and F08, though F09’s locations were evenly distributed throughout her area. Although F09 showed comparable evasive behavior to overall treatment activity, interesting anecdotal evidence arose regarding her tolerance to noise disturbance and logging practices during a spring denning season (T. Smith and D. Clayton, U.S. Forest Service, unpublished data). Timber activity within F09’s home range commenced in April 2012, with the objective of cutting pine and fir trees with subsequent helicopter yarding to remove the fallen logs. Within days after felling the trees, F09 chose a standing, live conifer in the middle of the logged unit to birth her kits in a natal den. Helicopter yarding began before we discovered the den site, with high decibel noise and frequent mechanical disturbance occurring adjacent to her den tree. Upon discovery, managers postponed helicopter operations until she moved her kits to a new site further away. Fisher F09 did not relocate her den for 2 weeks despite a constant flow of human activity occurring nearby. She eventually moved to a secondary (maternal) den outside the treated unit, where she successfully raised three kits past the weaning stage. Although exposure to short-term noise disturbance was not a study objective, this event indicates that even during a reproductively sensitive time, F09 did not react negatively to noise levels or sudden vegetation changes in her physical environment. Equally important, there were no adverse effects observed in kit development or care.

Interestingly, F09’s denning behavior contradicted the results of her overall disturbance response to treatments during the entire study period. In 2013 and 2014, F09 continued to den in commercially treated units, but only after treatments were completed; one den was used within 6 months after a unit had received treatment. Similarly, other female fishers selected den structures within treated units. Fisher F03 was observed to be denning on two separate occasions in noncommercial units and once in a commercially treated unit 4 years after completion. Fisher F01 denned on private land shortly after the landowner finished a large thinning project that removed a majority of conifers. Although our study did not analyze den selection factors in response to forestry practices, we recommend future research assess what habitat components are important to denning fishers as habitat quality changes.

We also investigated management intensity levels (commercial versus noncommercial treatments) and found no significant differences in fisher distance behavior between these activities. Analysis of locations found inside and outside the treatments in a temporal context did not lend any significance either, though the raw number of locations in commercial (*n* = 52) versus noncommercial (*n* = 63) areas were slightly lower. We speculate that because canopy cover change post treatment was minimal and optimal habitat was still retained in both treatment types, fishers did not discern any threats that would minimize use in these areas. Finally, we originally intended to use treatment duration as a variable of interest, but we were unable to obtain available data for statistical testing. We suggest future analyses consider treatment duration as a potential factor affecting fisher behavior.

When exploring other covariates affecting behavior, season and vegetation class were found to influence fisher distance to a pre-treated unit. We reason that this outcome is valid; because no change in habitat quality had yet taken place, other ecological pressures could account for variable fisher movement (e.g., seasonal food availability, predator evasion). Two variables of significance common to fisher’s post-treatment were season and treatment size. Fishers were observed to be further from treated units in fall and winter, and further from treated units that ranged from very small to medium in size (<12 ha). We speculate that larger units may have included suitable habitat to travel through and use, while the smaller units were avoided or circumnavigated if habitat had been reduced. Our season covariate was significant in both periods for all fishers, and the majority of units were treated in spring or summer (63% total). Curiously, fishers were closer to treatment areas at the time they were treated, but this result may have been due to an elimination of sampling points within a 30-day treatment period, lag effects of vegetation removal, or unrelated factors such as shifts in food availability.

We expected canopy cover to be the most important constituent influencing treatment response, especially given that multiple studies have cited it as a principal element crucial to fisher habitat use [5,35,64]. However, descriptions of canopy cover can be subjective and difficult to interpret, as measurement methods vary between studies [8,65]. Overhead canopy cover in our area was not manipulated in noncommercial units, though understory cover may have been reduced to a small degree. Commercial units, on the other hand, reduced canopy cover as was intended in the AFR objectives. Nevertheless, relative changes in canopy cover were minimal for treatments, with few units resulting in a reduction to <60% canopy cover following treatment. Therefore, we surmise that adequate overhead cover (>60%) was retained throughout most of the post-treatment home ranges, and a majority of fishers were likely not impacted by canopy cover changes.

Other studies corroborate our findings of tolerance and variability in fisher response to a managed landscape. One study speculated that fishers can tolerate a portion of their home range being treated as long as adjacent higher-quality habitat is present [46]. Indeed, we concur because none of our fishers shifted their home range to exclude treated areas. Most of our treated units had adequate canopy cover, and both commercial and noncommercial units continued to be utilized. In a different perspective, one study found proximity to contiguous mature forest was the highest predictor of fisher presence in managed landscapes [35], rather than canopy cover; however, it is important to note that these were industrially managed forests with a far higher management intensity than the AFR landscape. Our results are consistent as well, since overhead cover was not a critical element in fisher space use. However, another study stated that canopy closure was their most impacted feature resulting from management practices [25], but they mention that this element was only tested in select stands and not extrapolated to the size of a fisher home range. The authors also noted that short-term effects from mechanical treatments were mitigated by the retention of larger trees for fisher use [25]. Our study resulted in similar findings, as the AFR objectives thinned only small-stemmed trees and left larger trees and snags on the landscape, thus allowing fishers to continue using suitable habitat within their home ranges [45]. Finally, a camera survey study concluded that extractive activities (e.g., timber harvests for commerce or hazard tree removal) had no impact on fisher use and presence, and although restorative fuel reduction practices lowered fisher occupancy, fishers continued to use the areas for multiple purposes and did not alter home range placement [24]. Again, our fuel reduction practices had both positive and negative effects on fishers, but the treatments studied here did not deter fishers from using a majority of their home range habitat, as evidenced by a tolerance threshold distance to treatments well within their territories.

The interpretation of wildlife behavior, whether applying personality traits or using distance measurements to index disturbance effects, must be used with caution. An animal may leave an area due to a disturbance event, but that decision does not necessarily mean it was negatively impacted by the event [61]. Other factors, such as environmental conditions (e.g., weather), prey availability, competition, or predator presence, could have influenced fisher movements within their home ranges independent of treatment effects. We were unable to assess all variables in our study due to the sheer complexity of ecological relationships and problems associated with our GPS transmitters, specifically inaccurate spatial locations and missed fixes. These challenges were due to terrain interference, dense vegetation cover, and infrequent scheduling of points [66,67]. Additionally, our study had a low number of sampled individuals. A larger sample size of both sexes and better GPS-fix schedules would certainly correct some of our sampling issues.

## 5. Conclusions

The removal and reconfiguration of habitat elements important to fisher survival and persistence, in the interest of increased forest resiliency, continues to be a contentious topic [1,18]. The response of wildlife to management actions, especially those that are highly dependent on specific habitat conditions, should be considered before treatment applications begin. Fishers use a variety of structures at ground level and throughout multiple canopied-layers, and the reconfiguration of home range features can alter behavioral patterns for foraging, denning, and travel paths [35]. Indeed, the majority of our fishers had a weak negative association with treatments in general and many fisher locations were found at greater distances for post-treatment areas than pre-treatment areas. Results to determine which specific factors influenced a fisher to move away from treatment units were somewhat ambiguous, and it seemed that decisions varied based on individual tolerance. However, we presume it will be difficult to differentiate what is occurring at the individual level and how that equates to the population as a whole, especially given that management practices spanned across home ranges of several animals. Seasonal timing of treatments, though, came out as a significant factor when investigating variables of interest. Using limited operating periods (LOPs) is a good practice that we suggest be continued in timber management plans, where forest operations are restricted during crucial wildlife reproductive periods [68]. Use of LOPs has been successfully implemented for other species, such as the northern spotted owl (*Strix occidentalis*) and northern goshawk (*Accipiter gentilis*). Similar proposals have been developed for the fisher, though there is some disagreement in determining an effective buffer size for known denning structures, because of multiple-use trees and large home ranges [10].

Nevertheless, fundamental principles in fisher behavior can assist managers in making beneficial decisions regarding fuel modifications to forest stands while maintaining basic fisher habitat needs. In particular, the identification and preservation of specific tree species that take decades to form cavities (i.e., legacy trees) or large growth should take precedence when formulating plans for thinning projects. Legacy trees, snags, and hardwood components, used for denning and resting, can be retained in select stands where vegetation diversity and regeneration are predominant. The added benefits of retaining these components not only provides refugia sites for habitat-obligate species, but also sustains key ecological processes, such as soil stabilization, seedling protection, and nutrient recycling [46,69]. The configuration of mixed-forest species and structures is also essential as it creates a diverse array of resources for fishers to exploit, and historical evidence shows that fire severity through mixed-forests is lower in those areas and maintains heterogeneity [70]. We believe, then, that data gathered from our research coupled with future studies might reveal new methods in mitigating the loss of stand heterogeneity at finer scales while allowing larger manipulations to occur on the landscape with minimal impact to fisher space use.

## Figures and Tables

**Table 1:** Number of locations, collar acquisition rates, days monitored, and years monitored for individual fishers in the Ashland Watershed Study Area, Oregon, 2010–2017. All female fishers were recaptured and fitted with a replacement GPS-collar throughout the study period. However, no replacement of collars occurred among males. F: female fisher, M: male fisher.

Fisher ID	Locations	Acquisition Rate (%)	Days Monitored	Years Monitored
F01	213	31	148	2010–2014, 2016
F02	90	24	67	2010–2011
F03	360	26	258	2011–2017
F04	43	24	32	2010–2011
F06	82	68	56	2011–2012
F08	123	25	80	2012–2014, 2017
F09	263	50	167	2012–2013
M02	24	13	22	2010
M04	70	34	55	2014
M10	84	60	40	2014
Total	1352	32	925	7 years

**Table 2:** Home-range size, and number, size, and proportion of total treated units and mechanical-only-treated units per fisher home range in the Ashland Watershed Study Area, Oregon, 2010–2017. Mechanical treatments were applied in commercial and noncommercial units, which were combined for this table. F: female fisher, M: male fisher.

Fisher ID	Home-Range Size (km^2^)	# of Treated Units in Home Range	Size of All Treated Units (ha)	Proportion of the Size of All Treated Units to Home Range Size	# of Mechanically Treated Units in Home Range	Size of Mechanically Treated Units (ha)	Proportion of Mechanically Treated Units to Home Range
F01	9.81	11	96	0.10	8	76	0.08
F02	14.99	85	696	0.46	50	429	0.29
F03	13.89	70	478	0.34	39	316	0.23
F04	20.69	73	834	0.40	47	464	0.22
F06	34.14	81	978	0.29	54	597	0.18
F08	12.40	61	553	0.45	46	378	0.31
F09	7.99	40	431	0.54	29	272	0.34
M02	65.42	86	1025	0.16	60	643	0.10
M04	102.23	136	1369	0.13	83	853	0.08
M10	40.23	76	993	0.25	48	606	0.15
Average	32.18	72	745		46	463	

**Table 3:** Silviculture treatment types by number of units, size in hectares, mean area per treatment, and proportion within the managed and total watershed area, Ashland Watershed Study Area, Oregon, 2010–2017.

Treatment Type	No. of Units	Area Size (ha)	Area Mean (ha)	Proportion of Treatment to Managed Area	Proportion of Treatment to Watershed Area ^a^
Commercial ^b^	59	498	8.44 ± 7.22	0.362	0.0199
Noncommercial ^b^	63	640	10.15 ± 11.49	0.465	0.0256
Burn only ^c^	9	185	20.61 ± 22.50	0.134	0.0074
Untreated ^d^	7	53	7.62 ± 3.46	0.039	0.0021
Total	138	1376	--	1	0.0551

^a^ The Ashland Watershed Study Area totaled 24,970 ha. ^b^ Mechanically treated units were categorized as either commercial or noncommercial type. ^c^ Burn treatment category included under burns and pile burns only. ^d^ Untreated units were slated for treatment in the original management plan but were not implemented during our study.

**Table 4:** Pre- and post-treatment results for individual fishers using multi-response permutation procedure (MRPP) test, Kolmogorov–Smirnov (KS) test, and chi-squared independence test, Ashland Watershed Study Area, Oregon, 2010–2017. F: female fisher, M: male fisher.

		MRPP			KS		Chi-Square Test		
Fisher ID		Delta	A	*p*	D	*p*	χ^2^	df	*p*
F01	Pre	782	0.039	<0.001	0.280	<0.001	187.69	7	<0.001
	Post	807							
F02	Pre	1265	0.004	<0.001	0.127	<0.001	129.59	7	<0.001
	Post	847							
F03	Pre	940	0.001	<0.001	0.056	<0.001	78.49	7	<0.001
	Post	904							
F06	Pre	1752	0.002	<0.001	0.083	0.004	21.98	7	0.003
	Post	2041							
F08	Pre	1175	0.038	<0.001	0.344	<0.001	303.04	7	<0.001
	Post	1302							
F09	Pre	672	0.021	<0.001	0.176	<0.001	238.70	7	<0.001
	Post	827							
M02	Pre	3711	0.003	<0.001	0.221	0.021	10.56	7	0.159
	Post	4069							
M10	Pre	504	0.004	<0.001	0.167	<0.001	41.95	7	<0.001
	Post	4704							

**Table 5:** Multi-way ANOVA results for (**A**) pre-treatment and (**B**) post-treatment for pooled subset of fisher data analyzing factors that affect fisher distance to treatments in the Ashland Watershed Study Area, Oregon, 2010–2017. Fishers combined in the analysis include F01, F08, and F09.

(A)					
Pre-Treatment Variable	df	Sum of Squares	Mean Squares	F	*p*
Season	3	5.17 × 10^7^	17,241,944	41.2387	<0.001
Time of day	3	4.00 × 10^6^	1,332,588	3.1872	0.023
Treatment type	1	8.06 × 10^6^	80,645,488	19.2886	<0.001
Vegetation class	4	2.59 × 10^7^	6,484,672	15.5098	<0.001
Treatment size	4	5.76 × 10^6^	1,439,561	3.4431	<0.001
Canopy cover	3	2.14 × 10^7^	7,121,796	17.0337	<0.001
Error	3536	1.48 × 10^9^	418,101		
**(B)**					
**Post-Treatment Variable**	**df**	**Sum of Squares**	**Mean Squares**	**F**	***p***
Season	3	2.56 × 10^8^	85,197,703	161.9846	<0.001
Time of day	3	2.16 × 10^6^	720,643	1.3701	0.250
Treatment type	1	6.80 × 10^5^	679,606	1.2921	0.256
Vegetation class	4	7.63 × 10^7^	19,067,905	36.2534	<0.001
Treatment size	4	1.06 × 10^8^	26,457,317	50.3027	<0.001
Canopy cover	3	4.25 × 10^6^	1,416,785	2.6937	0.004
Error	3536	4.41 × 10^9^	525,962		

## Data Availability

Data available upon request from U.S. Forest Service biologists T.R.S. and R.D.C.
